# Alterations in Corpus Callosum Subregion Microstructure Associated With Visual Dysfunction in Individuals With Cerebral Visual Impairment

**DOI:** 10.1155/bn/3687804

**Published:** 2026-09-07

**Authors:** Marie Drottar, Chan-Mi Kim, Howard J. Cabral, Corinna M. Bauer

**Affiliations:** ^1^ Laboratory of Neuroimaging and Vision Science, Department of Radiology, Massachusetts General Hospital, Boston, Massachusetts, USA, harvard.edu; ^2^ Department of Radiology, Gordon Center for Medical Imaging, Massachusetts General Hospital, Harvard Medical School, Boston, Massachusetts, USA, harvard.edu; ^3^ Department of Biostatistics, Boston University School of Public Health, Boston, Massachusetts, USA, bu.edu; ^4^ Department of Ophthalmology, Harvard Medical School, Boston, Massachusetts, USA, harvard.edu

**Keywords:** cerebral (cortical) visual impairment (CVI), corpus callosum, CVI questionnaire, diffusion, tractography, visual perception

## Abstract

**Background:**

Cerebral/cortical visual impairment (CVI) is among the leading causes of visual impairment in children. Yet, identifying the neural correlates of CVI remains challenging due to the heterogeneous underlying etiologies and visual manifestations. While previous work has focused on intra‐hemispheric white matter connections, in this cross‐sectional observational study, we focus on the potential contribution of inter‐hemispheric white matter connections via the corpus callosum (CC) to the severity of visual dysfunction in individuals with CVI.

**Methods:**

Seven segments of the CC were isolated in 21 participants with CVI (10 M, 11 F, mean age: 20.19 years, 8.38 s.d., range: 10–44 years) and 26 controls (10 M, 16 F, mean age: 19.00 years, 5.82 s.d., range: 10–28 years) without a history of brain injury or visual impairment. Mixed models were used to investigate within‐ and between‐group differences in diffusion metrics. Associations between diffusion metrics of the CC segments and severity of visual dysfunctions across five domains determined by the CVI Questionnaire were calculated in an exploratory analysis within the CVI group.

**Results:**

Significantly lower fractional anisotropy (FA) and higher radial diffusivity (RD) measures were observed for isthmus and splenium, as well as higher mean diffusivity (MD) for the splenium in the CVI group compared with control following correction for multiple comparisons. In our exploratory analysis, the severity of impairments in object and face processing, impairments moving in space, and anxiety‐related behaviors were significantly negatively associated with diffusion properties of posterior midbody CC segments in the CVI group. Additional positive correlations between visual disinterest and AD of the rostrum were also observed.

**Conclusions:**

Our results demonstrate that while CVI is associated with atypical diffusion properties in the posterior portions of the CC, the severity of visual dysfunctions may be associated with underlying tissue composition of the anterior and mid‐posterior segments of the CC.

## 1. Introduction

Cerebral (cortical) visual impairment (CVI) represents the leading individual cause of visual impairment in many countries, affecting between 20% and 75% of students with moderate to severe visual impairment [1–3]. Notably, the current estimates of individuals with CVI and good visual acuity remains unclear [4, 5]. CVI is one of the potential sequalae of early injury, damage, malformation, or malfunctioning of the visual processing centers in the brain and can manifest as a spectrum of impairments in visual function and/or functional vision [6–10]. Due in part to its heterogeneity of presentation and underlying etiologies, there remain a number of unanswered questions related to the neural underpinnings of CVI and the potential for neuroplasticity in this population. While increasing efforts have been made to understand the underlying neural correlates of visual dysfunction associated with CVI [11–18], the role of the corpus callosum (CC) in CVI remains unclear.

Through both homotopic and heterotopic connections, the CC is integral to the interhemispheric integration of distributed neural circuits associated with motor, cognitive, and sensory functions [19–21]. The CC also mediates the transfer and integration of information across the left and right visual hemifields, a task central to visually guided behaviors such as reading and visual search. Notably, impairments in reading and visual search have often been reported in individuals with abnormal development or damage to the various subregions of the CC [22–26]. However, the association between the CC and degree of visual impairment and or difficulty with vision‐related tasks has not been investigated in individuals with CVI, representing a significant gap in knowledge. Given its central role in visual perception, a thorough quantitative investigation will provide much‐needed evidence supporting the potential relationship between atypical CC maturation and visual dysfunctions in individuals with CVI.

In the case of CVI, agenesis or thinning of the CC has been reported in qualitative and semi‐quantitative reports of neuroimaging findings in children and adolescents with CVI and related conditions, including CP [11, 27–30]. As the CC may be integral for supporting interhemispheric neuroplasticity following brain injury [31, 32], having a better understanding of how it may be affected in those with CVI due to early brain injury may provide invaluable insight into the potential for interhemispheric plasticity. Further, although the CC has already reached its peak fiber number at birth, CC myelination continues in a caudal–rostral fashion throughout childhood [33], supporting a potential mechanism for plasticity [31, 32, 34].

The CC displays regional variation in both the types and densities of fibers it contains. In general, there is a rostral–caudal gradient with higher density and lower diameter fibers in the anterior portion compared with the posterior portions, with increased density again in the splenium, which connects visual cortices [35]. Because of the regional variation in maturational profile of the CC [36], it is possible that early developmental brain injury affects axonal properties, including myelination, which may be different across each segment. Previous evidence suggests that the isthmus and splenium may be particularly affected in neurodevelopmental conditions, such as Williams syndrome and cerebral palsy [30, 37]. However, despite the potential role of the CC in visual perception, it is unclear whether aberrant callosal development contributes to the visual dysfunctions reported by individuals with CVI.

Thus, in this study, we characterized the regional profiles of diffusion properties across the CC in individuals with CVI and typically sighted controls to test the hypothesis that CVI is associated with significant differences in diffusivity and anisotropy in the posterior segments of the CC. As a secondary exploratory analysis, we used partial correlation analyses to test the hypothesis that diffusion properties reflecting axonal damage and dysmyelination (i.e., decreased AD and increased RD) are significantly associated with the severity of visual dysfunctions and challenges with the functional use of vision as determined by the Flemish CVI Questionnaire (FCVIQ) [38].

## 2. Methods

### 2.1. Participants

Twenty‐one participants with CVI (10 M, 11 F, mean age: 20.19 years, 8.38 standard deviation [s.d.], range: 10–44 years) and 26 controls (10 M, 16 F, mean age: 19.00 years, 5.82 s.d., range: 10–28 years) were enrolled in the study. Participants were recruited using convenience sampling through ties with the greater Boston area community and through connections with the CVI parent, clinical, and education communities across New England and surrounding states. Enrollment, testing, and MRI scanning were completed between November 2021 and November 2025. Generally, testing and scanning occurred on the same day or on consecutive days. Participants with CVI were previously diagnosed by their eye care or other medical professional prior to enrollment in the study based on a thorough examination of their ocular structures, medical history, and visual behaviors, including lower‐order visual functions and higher‐order visual perception [17, 39]. A diagnosis was made when the visual dysfunction was greater than could be expected based on ocular findings alone, in agreement with current guidance [40–42]. Inclusion criteria for the CVI group included: a clinical diagnosis of CVI, best corrected binocular visual acuity better than 20/70 Snellen (0–0.544 logMAR equivalent), and cognition sufficient to understand task instructions. Exclusion criteria included the following: aged < 10 or > 45 years, claustrophobia, presence of a metallic foreign body, presence of an auditory impairment that would restrict verbal communication with the research team during scanning, or the inability to lie still for periods of up to 10 min at a time without sedation, as this would affect quality of the MRI data. Additional exclusion criteria for the control group (including those born preterm) included a known history of an ocular disorder (including high grade retinopathy of prematurity), visual field restriction, visual acuity (or best corrected visual acuity) worse than LogMAR 0.0 (i.e., 20/20 Snellen), or any known neurologic condition. Participant details can be found in Table 1.

**Table 1:** CVI participant details.

ID	Age	Sex	Preterm	Logmar acuity	Oculomotor dysfunction	PVL	Suspected cause of CVI	Co‐occurring conditions
1	14	F	Yes	0.0969	None	No	In‐utero stroke	CP
2	25	F	No	0	Strabismus	No	Other, polymicrogyria	Apraxia
3	23	F	Yes	0.301	None	No	HI injury at birth	CP
4	20	F	No	0.544	None	No	Meningitis, occipital stroke in infancy	
5	12	F	No	0	Optic ataxia, CI, AI	No	Perinatal head injury, hypoglycemia	Possible dyslexia
6	20	F	No	0	None	No	Birth complications	
7	23	F	No	0.544	Nystagmus	No	Unknown, Low birth weight noted	ASD
8	21	M	No	0.301	None	No	Infantile seizure	
9	18	M	No	0	CI	No	Hypoxia at birth	Dyspraxia, dyscalculia, motor dyspraxia, ASD
10	10	M	Yes	0	Esotropia	Yes	Perinatal hypoxic brain injury	CP, hypotonia
11	17	M	No	0.0969	None	No	Anoxic trauma at 3 months	ASD, APD, dyspraxia, epilepsy
12	15	M	No	0	None	Yes	PVL	CP (spastic diplegia)
13	30	M	Yes	0.398	None	Yes	Premature birth, low birth weight, PVL	CP, dyslexia, dyspraxia, epilepsy
14	12	M	Yes	0.176	Corrected strabismus	Yes	Cystic PVL, IVH, premature birth	CP
15	10	M	Yes	0	Exotropia	No	Myopathy, delayed occipital myelination, ND1 and ND5 genetic disorder	History of seizures, dyslexia, dyscalculia
16	25	M	Yes	0	Strabismus	Yes	PVL, hydrocephalus	CP
17	27	M	No	0	CI	No	Birth complications	ID
18	12	F	Yes	0.477	Latent nystagmus, intermittent exotropia	Yes	Premature birth, PVL, respiratory distress	CP
19	31	F	No	0.301	Corrected strabismus, Hx amblyopia	No	Viral encephalitis	History of seizures, ID
20	15	F	No	0	Nystagmus	No	Birth complications related to multiple births	ASD, ADHD, ID
21	44	F	No	0		No	Occipital hemorrhage	

Abbreviations: AI: accommodative insufficiency, APD: auditory processing disorder; ASD: autism spectrum disorder; CI: convergence insufficiency, CP: cerebral palsy, Hx: history, IVH: intraventricular hemorrhage, PVL: periventricular leukomalacia.

Informed consent (or assent in the case of minors) was obtained from all participants prior to enrollment in accordance with ethical approval from the Massachusetts General Brigham Institutional Review Board and the Declaration of Helsinki.

### 2.2. Functional Vision

The Flemish Cerebral Visual Impairment questionnaire [38] was administered to 19 of the 21 (90.5%) participants with CVI (unavailable for CVI 3 and 21). In addition to the total number of items marked as difficult, scores were calculated for each factor and converted to percentages, namely object‐face processing, visual disinterest, clutter distance viewing, moving in space, anxiety‐related behavior [43, 44]. Thus, higher scores reflect a higher proportion of items marked as difficult.

### 2.3. MRI Acquisition

All MRI scanning was completed at the Boston University School of Medicine Center for Translational NeuroImaging. T1‐weighted MRI and diffusion MRI (dMRI) were acquired on a Philips 3T Elition X System with a 32‐channel phased array head coil. For each participant, T1‐weighted MRI was acquired using magnetization prepared rapid gradient echo (MPRAGE) sequence (repetition time=6.50 ms, echo time = 2.90 ms, flip angle = 8.00°, acquired voxel size = 1 mm iso, reconstructed voxel size = 0.469 × 0.469 × 1 mm, 170 contiguous slices). Diffusion data was acquired using multi‐shell diffusion MRI acquisition (repetition time = 6185.00 ms, echo time = 89.00 ms, b‐values = 0, 500 (6 directions), 1000 (15 directions), 2000 (15 directions), 3000 (60 directions), total diffusion directions = 96, gradient strength 45 mT/M, slew rate 200 mT/m/s, multiband factor of 4, 2 mm isotropic voxel size), and corresponding reverse‐encoded field maps.

### 2.4. Data Preprocessing

T1 data were processed in FreeSurfer version 7.4.2 using the recon‐all pipeline as described in detail elsewhere [45–49]. Fieldmaps for the diffusion data were concatenated into a single file and top‐up was performed followed by brain extraction of the B0 image. Eddy cuda version 10.2 was used to correct for susceptibility and eddy distortion as well as inter‐ and intra‐slice motion. For each participant, the b‐vectors were rotated accordingly.

### 2.5. Diffusion Tractography

**Figure 1:**
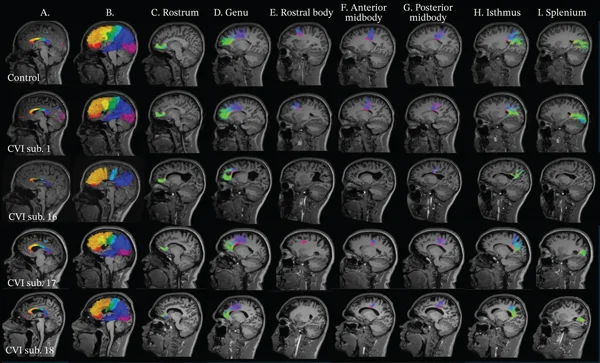
Representative examples of tractography of the CC segments for a control (top row) and four participants with CVI (subs 1, 16, 17, and 18 from the participant table). (A and B) Segments of the CC: red: rostrum, orange: rostral body, yellow: anterior midbody, green: posterior midbody, blue: isthmus, violet: splenium. Individual segments are shown in C through I and colors represent the directionality of the diffusion (green: rostral/caudal, blue: superior/inferior: red: left/right).

Following preprocessing, whole brain tractograms were reconstructed using MRtrix version 3.0.1 [50]. Data was processed by multishell multi‐tissue constrained spherical deconvolution and coregistered to the subject’s own b0. The whole brain tractogram was reconstructed with max length 250, anisotropy threshold of 0.06 (Otsu), with 1,000,000 tracts. Seven segments of the corpus callosum (CC1 rostrum, CC2 genu, CC3 rostral body, CC4 anterior midbody, CC5 posterior midbody, CC6 isthmus, and CC7 splenium) [51, 52] were auto reconstructed using TractSeg with anatomically constrained tractography in mrtrix (Figure 1) [53, 54]. Fractional anisotropy (FA), mean diffusivity (MD), axial diffusivity (AD), and radial diffusivity (RD) were sampled at 100 equally distributed points along each tract and averaged across the middle 80% of tracts to reduce the effects of crossing U‐fibers on diffusion outcomes [55]. The study team involved with MRI preprocessing and analyses were blinded to participant group. Further, diffusion analyses were automated as much as possible to reduce potential reviewer bias.

### 2.6. Statistical Analysis

Statistical analyses were completed using SAS Studio (SAS Institute, In. Cary, NC, United States). *t*‐tests were used to investigate group differences in age. Chi‐square was used to test for equal distribution of (1) males and females and (2) individuals born preterm across groups. Univariate tests for normality, including Q‐Q plots and visual inspection, were used to evaluate normal distribution of the data. Descriptive statistics (mean, s.d.) for the FCVIQ total and factor scores were calculated within the CVI group. A repeated‐measures analysis was used to investigate potential differences in FCVIQ factor scores in the group of participants with CVI, adjusting for the potential effects of age, sex, and preterm birth. Within‐group differences in diffusion outcomes across the CC segments were investigated through a series of repeated mixed‐models (i.e., two (group) by seven (CC segment)) adjusting for the potential effects of age, sex, and preterm birth. Initially, three‐way interaction effects of group∗CC segment∗preterm birth were included in the model. As none of the interaction terms involving preterm birth were significant, the models were re‐run with only the group∗CC interaction term and with preterm birth as a fixed effects covariate along with age and sex. Similarly, where the group∗CC segment interaction term was not significant, the model was re‐run collapsing data across both groups. In all cases, where the interaction term was significant, within group differences across segments were explored through Tukey–Kramer post hoc analyses. This process was repeated for each diffusion measure (i.e., FA, MD, AD, and RD). In our exploratory analysis investigating the association between FCVIQ and diffusion outcomes, Spearman partial correlations (adjusting for age, preterm birth, and sex) were used to investigate the relationship between CC diffusion outcomes and functional vision as indicated by FCVIQ total and factor scores. As the partial correlation analysis was exploratory without *a priori* defined hypotheses, and to avoid increasing potential Type II error and excluding clinically relevant associations [56], multiple comparisons correction was not conducted for this portion of the study. We used Spearman partial correlations as the FCVIQ factor scores are not continuous variables. For all analyses, *p* < 0.05 was chosen as the critical threshold for representing statistically significant effects.

## 3. Results

### 3.1. Participants

An equal number of males and females were in each group (chi − square = 0.3985, *p* = 0.5279) and the CVI and control groups did not differ significantly in age (*t* = 0.57, *p* = 0.5690). There were more individuals born preterm in the CVI group (*n* = 8, 38%) than the control group (*n* = 4, 15%); however, this did not reach statistical significance (chi − square = 3.15, *p* = 0.0759).

### 3.2. FCVIQ Factor Analysis

**Figure 2:**
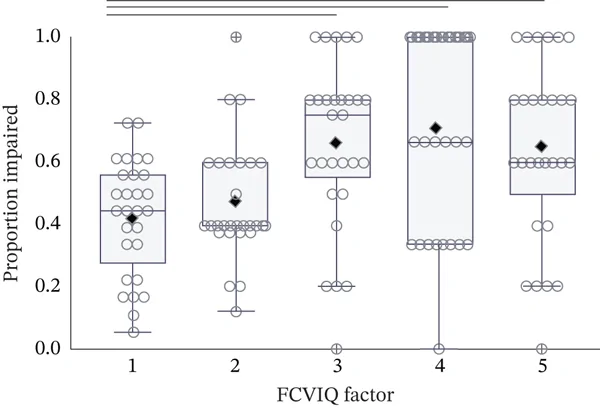
Box plots highlighting the distribution of the FCVIQ factor scores within the CVI group. The mean score is represented by the black dot and the median score by the horizontal line. Data from each participant is plotted with an open circle. Horizontal lines at the top reflect post‐hoc significant differences based on the repeated measures analysis adjusting for age, sex, and preterm birth. Factor 1: Object and face processing impairments, Factor 2: Visual disinterest, Factor 3: Impairments in clutter and distance viewing, Factor 4: Impairments moving in space, and Factor 5: Anxiety‐related behaviors.

We observed a significant overall effect of FCVIQ factor (F(4, 72) = 6.39, *p* = 0.0002), but no significant effects of age, sex, or preterm birth were observed (*p* > 0.05). Post‐hoc analysis indicated that scores for object and face processing impairments (Factor 1) were lower than for clutter and distance viewing impairment (Factor 3) (*t* = −3.93, *p* = 0.0002, adj.*p* = 0.00017), impairments moving in space (Factor 4) (*t* = −3.96, *p* = 0.0002, adj *p* = 0.0016), and anxiety‐related behaviors (Factor 5) (*t* = −3.40, *p* = 0.0011, adj. *p* = 0.0094) after adjusting for the potential confounds of age and sex (Figure 2).

Additionally, while visual disinterest (Factor 2) scores were significantly lower than clutter and distance viewing impairment (Factor 3) (*t* = −2.67, *p* = 0.0093, adj.*p* = 0.0684), impairments moving in space (Factor 4) (*t* = −2.70, *p* = 0.0087, adj *p* = 0.0644), and anxiety‐related behaviors (Factor 5) (*t* = −2.14, *p* = 0.0357, adj.*p* = 0.2146), these did not survive correction for multiple comparisons. Note that higher scores indicated greater levels of reported difficulty. There were no significant differences between Factors 3, 4, and 5. The results remained the same after removing age, sex, and preterm birth from the model. See Figure S1 for individual participant responses and Table S1 for details.

### 3.3. CC Subregion Analysis

**Figure 3:**
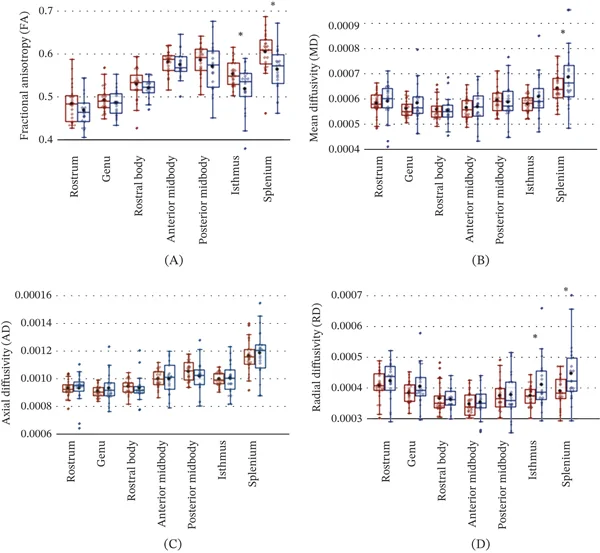
Box plots highlighting the within and between‐group differences in FA (A), MD (B), AD (C), and RD (D) for each segment of the CC. For each measure, the black dot reflects the within‐group mean and the horizontal line reflects the median. Control data is shown in red, CVI data is shown in blue. Asterisks reflect significant group differences based on the repeated measures analyses.

Mixed models were used to evaluate regional differences in CC FA (Figure 3a). No significant interaction between group∗CC segment∗preterm status was observed. Thus, the three‐way interaction term was removed from the model. Significant fixed effects for group (F(1, 43) = 4.79, *p* = 0.0341), segment (F(6, 264) = 108.73, *p* < 0.0001), preterm birth (F(1, 264) = 9.12, *p* = 0.0028, and a significant interaction effect between group and segment (F(6,264) = 2.63, *p* = 0.0172) were observed. No significant effects of sex were observed (*p* = 0.9538). Post‐hoc analysis followed by Tukey–Kramer correction for multiple comparisons revealed significant increases in FA in a caudal to rostral fashion (i.e., between both the rostrum and genu and the rostral body, anterior midbody, posterior midbody, isthmus, and splenium) with a decrease at the isthmus, which was significantly lower than the anterior midbody, posterior midbody, and splenium. These effects were seen in both groups. Additionally, simple effect comparisons between groups revealed significantly lower FA in the CVI group for the isthmus (t(264) = −2.98, adj.*p* = 0.0031) and splenium (t(264) = −3.63, *p* = 0.0003). See Tables S2 and S3 for full details of simple effects for CC segment and group, respectively.

Mixed models were used to evaluate regional differences in CC MD (Figure 3b). No significant interaction between group∗CC segment∗preterm status was observed. Thus, the three‐way interaction term was removed from the model. Significant fixed effects for segment (F(6, 264) = 42.31, *p* < 0.0001), and with a significant interaction effect between group and segment (F(6,264) = 2.74, *p* = 0.0133). No significant effects of group (*p* = 0.3298), sex (*p* = 0.2835), or preterm birth (*p* = 0.2327) were observed. In both groups, a general U‐shaped pattern was observed, with lower MD values in the rostral body and anterior midbody and significantly higher MD for the splenium. Simple effect comparisons between groups revealed significantly higher MD in the splenium following correction for multiple comparisons (t(264) = 2.46, *p* = 0.0145). See Tables S4 and S5 for full details of simple effects for CC segment and group, respectively.

Mixed models were used to evaluate regional differences in CC AD (Figure 3c). No significant interaction between group∗CC segment∗preterm status was observed. Thus, the three‐way interaction term was removed from the model. Similarly, we did not observe a significant interaction effect between group and segment (F(6,264) = 1.21, *p* = 0.2988), so the interaction term was removed from the model and it was re‐run collapsing across groups. A significant effect of segment was observed (F(6, 270) = 98.07, *p* < 0.0001), but group (*p* = 0.7384), sex (*p* = 0.0749), and preterm birth (*p* = 0.8916) did not contribute significantly to the model. Across the combined groups, a general rostral to caudal increase in AD was observed. Significantly higher AD was observed for the splenium in particular (*p* < 0.0001 for all comparisons). See Table S6 for details of simple effects for segment differences across groups.

Mixed models were used to evaluate regional differences in CC RD (Figure 3d). No significant interaction between group∗CC segment∗preterm status was observed. Thus, the three‐way interaction term was removed from the model. Significant fixed effects for segment (F(6, 264) = 23.10, *p* < 0.0001) and a significant interaction effect between group and segment (F(6,264) = 3.81, *p* = 0.0012) were observed. No significant effects of group (*p* = 0.1604), sex (*p* = 0.4972), or preterm birth (*p* = 0.0603) were observed. In both groups, a general U‐shaped pattern was observed, with lower RD values in the rostral body, anterior midbody, and posterior midbody, as well as significantly higher RD for the rostrum and splenium. Simple effect comparisons between groups revealed significantly higher RD in the isthmus (t(264) = 2.21, adj.*p* = 0.0281) and the splenium (t(264) 3.35, adj.*p* = 0.0009) following correction for multiple comparisons. See Tables S7 and S8 for full details of simple effects for CC segment and group, respectively.

### 3.4. Association With Visual Behaviors

As an exploratory analysis, within the CVI group, we used partial Spearman correlations adjusting for the effects of age, sex, and premature birth to investigate the relationship between integrity metrics of the CC subdivisions and visual dysfunctions impacting daily life as reported via the FCVIQ. See Table S9 for details.

**Figure 4:**
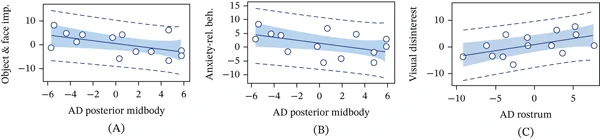
Partial correlation plots of the significant partial correlations between AD of posterior midbody of the CC and FCVIQ scores for (A) face and object processing impairments and (B) anxiety‐related behaviors and (C) between AD of the rostrum and visual disinterest within the CVI group adjusting for age, sex, and premature birth. The dotted lines represent the 95% prediction limit, the shared area shows the 95% confidence limit, and the solid line represents the partial correlation coefficient.

**Figure 5:**
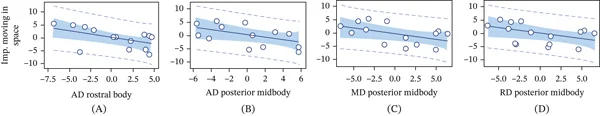
Partial correlation plots of the association between FCVIQ scores for impairments moving in space (Factor 4) and diffusion properties of the rostral body (A) and posterior midbody (B–D) within the CVI group adjusting for age, sex, and premature birth. The dotted lines represent the 95% prediction limit, the shared area shows the 95% confidence limit, and the solid line represents the partial Spearman correlation coefficient.

Object and face processing impairments were significantly negatively correlated with AD of the posterior midbody (r = −0.5725, *p* = 0.0257) (Figure 4a). Anxiety‐related behaviors were significantly negatively correlated with AD of the posterior midbody (r = −0.5249, *p* = 0.03445) (Figure 4b). Visual disinterest was significantly positively correlated with AD of the rostrum (r = 0.8582, *p* = 0.0277) (Figure 4c). Impairments moving in space were significantly negatively correlated with AD of the rostral body (r = −0.6129, *p* = 0.0341), posterior midbody (r = −0.5414, *p* = 0.0371), MD of the posterior midbody (r = −0.5286, *p* = 0.0428), and RD of the posterior midbody (r = −0.5323, *p* = 0.0411) (Figure 5).

## 4. Discussion

This study investigated the potential differences in diffusion microstructure of the CC subdivisions in CVI as compared with typically developing and sighted controls. The association of CC diffusion measures and extent of visual difficulties based on the Flemish CVI Questionnaire was also investigated in an exploratory analysis within the CVI group.

Participants with CVI included in our study showed fewer challenges on items related to object and face processing and visual disinterest and significantly more challenges in clutter and distance viewing, impairments moving in space, and anxiety‐related behaviors [43, 44].

Relatively similar patterns of diffusion differences across the CC segments were observed across both groups. Specifically, FA generally showed a general rostral to caudal increase, while diffusivity measures tended to be higher at either end within both CVI and control groups. A few notable significant group∗segment interactions were also present, with the CVI group having significantly lower FA and higher RD in the isthmus and splenium, as well as higher MD in the splenium in our post‐hoc analysis. These changes are consistent with freer diffusion within the white matter, particularly perpendicular to the fiber orientation, suggestive of greater water content related to reduced axonal density, demyelination, and/or a lack of myelinated fibers [57–60]. Similar patterns of callosal diffusion changes have been observed in survivors of stroke [32] and in adults born preterm with low birth weight [61, 62].

The majority of the differences in callosal diffusion metrics we observed between control and CVI groups were in the posterior segments (i.e., isthmus and splenium). Previous evidence suggests that the posterior CC is particularly vulnerable to injury and secondary effects following preterm birth [63–67]. The CC demonstrates regional variability in fiber composition and architecture, development of myelin, and morphometric growth rate, which may contribute to the regional susceptibility observed in CVI. While the CC continues to develop into adulthood [68–71], myelination begins rapidly in the first year of life, with higher rates in the splenium, which connects multiple occipital and parietal regions implicated in visual processing, as compared with the genu [72]. Notably, the splenium also exhibits more prolonged morphological development during adolescence as compared with the genu, which is among the earliest CC segment to develop [65, 70]. These periods of heightened myelination and axonal growth support the prolonged potential for plasticity. However, as windows of plasticity are also windows of heightened vulnerability [73], preterm birth or injury early in life may have prolonged consequences on myelination and axonal development of the CC and other structures [74]. Animal models of early hypoxic injury provide evidence for the long‐term reduction in myelination of the callosal axons [75]. Similarly, here, we observed significant increases in RD in the splenium and isthmus along with decreased FA, consistent with a lack of myelinated axons and reduced axonal density in CVI, which likely impact interhemispheric communication of both lower and higher order visual sensory areas. Follow‐up histopathological studies are needed to directly monitor the potential for dysmyelination or axonal injury in CVI.

In this study, although we did not observe any significant interaction effects with preterm birth, premature birth did contribute significant amounts of variance to FA measures. Infants born preterm are particularly vulnerable to PVL or other complications related to encephalopathy of prematurity [76], which may impact ongoing developmental processes of the CC [77]. For example, callosal fibers enter the subplate during the late second through early third trimester, the period of highest risk for PVL. During this time, injury may affect axon guidance and the formation of inter‐hemispheric connections. Similarly, during the late third trimester, the callosal fibers are entering the cortex [78], suggesting that the impact on the CC may depend partially on gestational length and/or age at time of injury [67]. In the present study, we did not have detailed information related to gestational age at birth and were unable to further evaluate effects of gestational length or age at time of injury on CC diffusion and the relationship with visual dysfunction; however, this would be an important topic to investigate in future studies.

Interhemispheric connections of the occipital cortex are primarily connected via the splenium of the CC, with a dorsal to ventral organization [20], aiding in a unified perception of visual information across the left and right hemifields [31]. Here, we report significantly lower FA and higher RD in splenium in our group of participants with CVI compared with controls. Together these results suggest a lack of myelinated fibers and lower axon density in this region. Contrary to what we anticipated, diffusion properties of the splenium did not contribute to the severity of reported visual dysfunctions in daily life as measured by the FCVIQ, it is possible that they are linked with other aspects of vision that were not evaluated as part of this study, such as functional vision, midlevel vision, or quantitative measures of visual perceptual behaviors such as motion detection, which may be associated with splenium integrity based on studies in patients with agenesis of the CC, hemianopia, or isolated splenium infarcts [31, 79–82]. Additional studies are needed to explore this possibility in individuals with CVI.

Here, the majority of the observed correlations in our exploratory analysis involved the posterior midbody. Specifically, AD and RD of the posterior midbody were weakly but significantly negatively correlated with FCVIQ scores related to object and face processing impairments, visual disinterest, and impairments moving in space. In other words, greater challenges in each category of visual behavior, as indicated by higher FCVIQ scores, were associated with lower diffusivity measures. In the case of AD, lower values reflect greater diffusion along the length of the axon (i.e., parallel to the axonal direction) consistent with pathophysiological measures of axonal damage [60, 83]. Thus, the negative correlations are consistent with our anticipated results: that greater CC injury is associated with more severe visual dysfunctions. However, the negative correlations with MD and RD were not anticipated. Generally, lower RD (i.e., reduced diffusion perpendicular to the fiber orientation) is thought to reflect greater myelination [59, 60]. However, RD is a nonspecific measure [84, 85] and diffusion perpendicular to the fiber orientation reflects not only myelination, but is also influenced by edema [86, 87], axonal packing [88, 89], and chronic axonal loss [90]. Further, the presence of crossing fibers may also restrict diffusion [91, 92]. Seeing as the participants in our study were all in the chronic stage of injury, it is less likely that the edema associated with the brain’s initial inflammatory response [93] was driving the diffusivity findings. Thus, it is more plausible that the observed negative association with RD and FCVIQ outcomes is associated with greater axonal packing/density, which has been noted in patients with hydrocephalus before surgery [94]. Additional studies using more advanced multicompartment diffusion modeling [95] and/or inter/extracellular T2 relaxation measures are required to further parse out these potentialities in vivo. However, as diffusion‐based and myelin water fraction provide indirect imaging markers, ex vivo histopathological studies of the CC from older children and adults who had CVI will need to be conducted to verify these proposed mechanisms and long‐term neural changes in this population.

The CC plays a major role in the integration of information between hemispheres. In the case of vision, this is integral for performing tasks such as visual selective attention and reading, both of which require attending to stimuli in both left and right hemifields. Indeed, difficulties with visual selective attention have been reported in individuals with agenesis of the CC due to difficulty reorienting to stimuli across hemifields [22]. While the precise mechanisms remain unknown, it is thought that the CC moderates interhemispheric communication in part via inhibitory interneurons [96, 97]. For example, combined evidence from patients with mirror movements in CP and from paired pulse transcranial magnetic stimulation (TMS) studies suggest that the main role of the CC may be inhibition of crosstalk between hemispheres. As such, reduced integrity of the CC, as is seen in patients with CP [30], may result in a reduction in the inhibition of the opposite hand leading to mirror movements—a form of maladaptive plasticity. In the case of CVI, the impact of CC plasticity on interhemispheric communication has yet to be investigated, although preliminary evidence suggests that impaired visual selective attention may be associated with a lack of inter‐hemispheric inhibition as reflected by greater functional connectivity [98]. Thus, it is possible that aberrant structural development results in compensatory increases in neural response profiles during challenging tasks [99]. This will need to be investigated further in future research.

This study has a number of limitations. First, we were limited in our recruitment by a relatively small sample size in part due to the relative rarity of a CVI diagnosis in adolescents and young adults as compared with other visual impairments and neurodevelopmental conditions. This was particularly true for the exploratory partial correlation analysis within the CVI group. Nevertheless, we were able to achieve power between 0.41 and 0.62 for each of the observed significant partial correlations with high effect sizes (i.e., over 0.35), based on post‐hoc power analysis in G∗Power. Additional studies with a sample size of at least 48 participants with CVI would be sufficient for providing a moderate effect size of 0.25% and 80% power. We were also unable to match our participants born preterm in the control and CVI groups based on gestational age. Further studies are needed to more fully distinguish between effects related to CVI and those related to preterm birth. Finally, because of convenience sampling, there may be participant bias and caution is advised when applying the findings to infants or younger children with CVI as well as to those who may have co‐occurring significant neurodevelopmental delays who may require sedation to be scanned, as these individuals did not meet inclusion criteria for this study. Nevertheless, as this study is among the first to evaluate the potential role of the CC in CVI, the findings represent a meaningful contribution to the literature and provide impetus for further research. There are also some limitations related to the methodology we used, specifically, we implemented a self‐ or proxy‐report of visual behaviors in daily life rather than empirical assessments of vision in order to include a broader spectrum of visual dysfunctions. We were also limited to cross‐sectional data, so it remains unclear whether those with CVI demonstrate an altered developmental trajectory of the CC, which is known to continue to develop throughout adolescence. It is also possible that neuroplastic mechanisms enable strengthened inter‐hemispheric connections through the anterior or posterior commissures in those with CVI. However, these bundles were not included in the current analysis, but warrant investigation in future studies as strengthening of the commissures has been noted in a case report of an individual with agenesis of the CC [80]. Because of the exploratory nature of the partial correlation analysis, the results may suffer from Type I error and will need to be interpreted with caution until they can be replicated in a secondary sample. This study is also limited to include only MRI diffusion data, however more advanced approaches, such as myelin water fraction imaging or pixel‐based analysis could provide additional information regarding the tissue composition and fiber architecture. Despite these limitations, the between‐ and within‐group effects related to integrity of the CC represent a significant contribution to the body of literature pertaining to the long‐term neurodevelopmental consequences of early brain injury in those with CVI.

## 5. Conclusion

Our results demonstrate that CVI may be associated with an atypical regional white matter diffusion profile within specific segments of the CC, which may reflect underlying differences in tissue composition. Indeed, the genu, isthmus, and splenium all showed significant differences in diffusion properties as compared with the control group. These CC segments connect cortical regions implicated in the processing of visual and visual perceptual information including visual, parietal, temporal, and frontal cortices. In our exploratory correlation analysis, diffusion properties of CC segments, in particular the posterior midbody, were associated with visual disinterest, anxiety‐related behavior and impairments in object and face processing, clutter and distance viewing, and moving in space. The aberrant regional CC diffusion properties reported herein provides insight into the neural correlates of the daily challenges in functional vision reported by those with CVI.

The findings support the need to evaluate vision use in children with injury to the developing CC or with conditions that affect callosal development, such as preterm birth, PVL, and cerebral palsy. The FCVIQ and other questionnaires, such as the Higher Visual Function Skills Inventory (or Top 11 items thereof) [5], are increasingly used to screen patients for impairments in functional use of vision who should receive a full multidisciplinary assessment for CVI. Future studies are required to verify the exploratory findings of this study in a larger sample of individuals with CVI. Given the exploratory nature of these associations, they will need to be replicated and should be interpreted with caution. Additionally, further research is needed to determine the potential role of CVI etiology or subtyping as well as the potential longitudinal changes in diffusion outcomes and visual abilities in children with CVI that occur as a result of neurodevelopmental plasticity or following targeted interventions.

**Nomenclature**
AD: axial diffusivity
CC: corpus callosum
CP: cerebral palsy
CVI: cerebral visual impairment
FA: fractional anisotropy
FCVIQ: Flemish Cerebral Visual Impairment Questionnaire
MD: mean diffusivity
RD: radial diffusivity

## Funding

This study was supported by the National Institutes of Health, 10.13039/100000002, EY030877.

## Ethics Statement

The study was approved by the Investigative Review Board at Massachusetts General Brigham, Boston, MA (2022P000532, 2019P003229) and conducted in accordance with the Declaration of Helsinki for research involving humans.

## Consent

Written informed consent was obtained from all participants or a parent/legal guardian along with participant assent (in the case of a minor).

## Conflicts of Interest

The authors declare no conflicts of interest.

## Supporting information


**Supporting Information** Additional supporting information can be found online in the Supporting Information section. Supporting Information. All supplemental information is provided in Supplemental_tables.docx. Specifically, individual participant responses for the FCVIQ domains are shown in Figure S1. Within‐group repeated measures results for FCVIQ factors with Tukey–Kramer adjustment for multiple comparisons are provided in Table S1. Simple effects pertaining to within or between group differences in diffusion outcomes for CC segments are detailed in Tables S2, S3, S4, S5, S6, S7, and S8. Table S9 provides details for the Spearman partial correlations for each CC segment and FCVIQ factor in the CVI group.

## Data Availability

De‐identified data is available following a data use agreement and approval from the local institutional review board.
