# Supplementary material for: Alterations in Corpus Callosum Subregion Microstructure Associated With Visual Dysfunction in Individuals With Cerebral Visual Impairment
Source: Behav Neurol. 2026 Sep 7;2026:3687804. doi: 10.1155/bn/3687804 (PMC13548053; doi:10.1155/bn/3687804)
Supplement: Supplementary file 1 [file BN-2026-3687804-s001.docx]

**Supplemental Figure 1.** Box plots highlighting the distribution of the FCVIQ factor scores for each participant with CVI for which the FCVIQ was available. Factor 1: Object and face processing impairments (blue), Factor 2: Visual disinterest (red), Factor 3: Impairments in clutter and distance viewing (green), Factor 4: Impairments moving in space (brown), and Factor 5: Anxiety-related behaviours (purple). Participant numbers correspond to Table 1.

**Supplemental Table 1.** Within-group repeated measures results for FCVIQ factors with Tukey Kramer adjustment for multiple comparisons adjusting for potential effects of age, sex, and preterm birth. Factor 1: object-face processing impairments, Factor 2: visual disinterest, Factor 3: clutter & distance viewing impairments, Factor 4: Impairments moving in space, Factor 5: anxiety related behavior.

| **FCVIQ Factors being compared** | | **Estimate** | **Standard Error** | **DF** | **t Value** | **Pr > \|t\|** | **Adj p.** |
| --- | --- | --- | --- | --- | --- | --- | --- |
| 1 | 2 | -0.08757 | 0.06946 | 72 | -1.26 | 0.2115 | 0.7158 |
| 1 | 3 | -0.2731 | 0.06946 | 72 | -3.93 | 0.0002 | 0.0017* |
| 1 | 4 | -0.2749 | 0.06946 | 72 | -3.96 | 0.0002 | 0.0016* |
| 1 | 5 | -0.2363 | 0.06946 | 72 | -3.40 | 0.0011 | 0.0094* |
| 2 | 3 | -0.1855 | 0.06946 | 72 | -2.67 | 0.0093 | 0.0684 |
| 2 | 4 | -0.1873 | 0.06946 | 72 | -2.70 | 0.0087 | 0.0644 |
| 2 | 5 | -0.1487 | 0.06946 | 72 | -2.14 | 0.0357 | 0.2146 |
| 3 | 4 | -0.00175 | 0.06946 | 72 | -0.03 | 0.9799 | 1.0000 |
| 3 | 5 | 0.03684 | 0.06946 | 72 | 0.53 | 0.5975 | 0.9839 |
| 4 | 5 | 0.03860 | 0.06946 | 72 | 0.56 | 0.5802 | 0.9809 |

**Supplemental Table 2.** Mixed repeated measures results for FA differences across CC segments collapsed across groups followed by Tukey Kramer correction for multiple comparisons.

| **CC segments being compared** | | **Estimate** | **Standard Error** | **DF** | **t Value** | **Pr > \|t\|** | **Adj p** |
| --- | --- | --- | --- | --- | --- | --- | --- |
| Rostrum | Genu | -0.01196 | 0.005890 | 264 | -2.03 | 0.0433 | 0.3979 |
| Rostrum | Rostral Body | -0.04553 | 0.006042 | 264 | -7.54 | <.0001 | <.0001 |
| Rostrum | Anterior Midbody | -0.09902 | 0.005986 | 264 | -16.54 | <.0001 | <.0001 |
| Rostrum | Posterior Midbody | -0.1000 | 0.005890 | 264 | -16.98 | <.0001 | <.0001 |
| Rostrum | Isthmus | -0.05835 | 0.005890 | 264 | -9.91 | <.0001 | <.0001 |
| Rostrum | Splenium | -0.1061 | 0.005890 | 264 | -18.00 | <.0001 | <.0001 |
| Genu | Rostral Body | -0.03357 | 0.005996 | 264 | -5.60 | <.0001 | <.0001 |
| Genu | Anterior Midbody | -0.08706 | 0.005941 | 264 | -14.65 | <.0001 | <.0001 |
| Genu | Posterior Midbody | -0.08805 | 0.005845 | 264 | -15.07 | <.0001 | <.0001 |
| Genu | Isthmus | -0.04639 | 0.005845 | 264 | -7.94 | <.0001 | <.0001 |
| Genu | Splenium | -0.09409 | 0.005845 | 264 | -16.10 | <.0001 | <.0001 |
| Rostral Body | Anterior Midbody | -0.05349 | 0.006082 | 264 | -8.80 | <.0001 | <.0001 |
| Rostral Body | Posterior Midbody | -0.05449 | 0.005996 | 264 | -9.09 | <.0001 | <.0001 |
| Rostral Body | Isthmus | -0.01283 | 0.005996 | 264 | -2.14 | 0.0333 | 0.3328 |
| Rostral Body | Splenium | -0.06052 | 0.005996 | 264 | -10.09 | <.0001 | <.0001 |
| Anterior Midbody | Posterior Midbody | -0.00099 | 0.005941 | 264 | -0.17 | 0.8671 | 1.0000 |
| Anterior Midbody | Isthmus | 0.04067 | 0.005941 | 264 | 6.85 | <.0001 | <.0001 |
| Anterior Midbody | Splenium | -0.00703 | 0.005941 | 264 | -1.18 | 0.2377 | 0.8999 |
| Posterior Midbody | Isthmus | 0.04166 | 0.005845 | 264 | 7.13 | <.0001 | <.0001 |
| Posterior Midbody | Splenium | -0.00604 | 0.005845 | 264 | -1.03 | 0.3027 | 0.9460 |
| Isthmus | Splenium | -0.04770 | 0.005845 | 264 | -8.16 | <.0001 | <.0001 |

**Supplemental Table 3.** Mixed repeated measures results for between-group differences in FA across CC segments followed by Tukey-Kramer correction for multiple comparisons.

| **CC Segment** | **Estimate** | **Standard Error** | **DF** | **t Value** | **Adj p** |
| --- | --- | --- | --- | --- | --- |
| Rostrum | -0.01262 | 0.01099 | 264 | -1.15 | 0.2519 |
| Genu | -0.00555 | 0.01088 | 264 | -0.51 | 0.6103 |
| Rostral Body | -0.00966 | 0.01118 | 264 | -0.86 | 0.3884 |
| Anterior Midbody | -0.00567 | 0.01107 | 264 | -0.51 | 0.6092 |
| Posterior Midbody | -0.01326 | 0.01088 | 264 | -1.22 | 0.2239 |
| Isthmus | -0.03246 | 0.01088 | 264 | -2.98 | 0.0031 |
| Splenium | -0.03949 | 0.01088 | 264 | -3.63 | 0.0003 |

**Supplemental Table 4.** Mixed repeated measures results for MD differences across CC segments collapsed across groups followed by Tukey Kramer correction for multiple comparisons.

| **CC segment** | **CC segment** | **Estimate** | **Standard Error** | **DF** | **t Value** | **Pr > \|t\|** | **Adj p** |
| --- | --- | --- | --- | --- | --- | --- | --- |
| Rostrum | Genu | 0.000013 | 7.587E-6 | 264 | 1.67 | 0.0960 | 0.6364 |
| Rostrum | Rostral Body | 0.000023 | 7.789E-6 | 264 | 2.90 | 0.0040 | 0.0601 |
| Rostrum | Anterior Midbody | 0.000016 | 7.716E-6 | 264 | 2.09 | 0.0379 | 0.3637 |
| Rostrum | Posterior Midbody | -7.66E-6 | 7.587E-6 | 264 | -1.01 | 0.3138 | 0.9516 |
| Rostrum | Isthmus | -0.00001 | 7.587E-6 | 264 | -1.32 | 0.1882 | 0.8425 |
| Rostrum | Splenium | -0.00008 | 7.587E-6 | 264 | -10.66 | <.0001 | <.0001 |
| Genu | Rostral Body | 9.949E-6 | 7.727E-6 | 264 | 1.29 | 0.1990 | 0.8572 |
| Genu | Anterior Midbody | 3.425E-6 | 7.655E-6 | 264 | 0.45 | 0.6549 | 0.9994 |
| Genu | Posterior Midbody | -0.00002 | 7.528E-6 | 264 | -2.70 | 0.0074 | 0.1020 |
| Genu | Isthmus | -0.00002 | 7.528E-6 | 264 | -3.01 | 0.0028 | 0.0444 |
| Genu | Splenium | -0.00009 | 7.528E-6 | 264 | -12.43 | <.0001 | <.0001 |
| Rostral Body | Anterior Midbody | -6.52E-6 | 7.836E-6 | 264 | -0.83 | 0.4058 | 0.9814 |
| Rostral Body | Posterior Midbody | -0.00003 | 7.727E-6 | 264 | -3.92 | 0.0001 | 0.0022 |
| Rostral Body | Isthmus | -0.00003 | 7.727E-6 | 264 | -4.22 | <.0001 | 0.0007 |
| Rostral Body | Splenium | -0.00010 | 7.727E-6 | 264 | -13.40 | <.0001 | <.0001 |
| Anterior Midbody | Posterior Midbody | -0.00002 | 7.655E-6 | 264 | -3.10 | 0.0021 | 0.0342 |
| Anterior Midbody | Isthmus | -0.00003 | 7.655E-6 | 264 | -3.41 | 0.0007 | 0.0131 |
| Anterior Midbody | Splenium | -0.00010 | 7.655E-6 | 264 | -12.67 | <.0001 | <.0001 |
| Posterior Midbody | Isthmus | -2.35E-6 | 7.528E-6 | 264 | -0.31 | 0.7549 | 0.9999 |
| Posterior Midbody | Splenium | -0.00007 | 7.528E-6 | 264 | -9.73 | <.0001 | <.0001 |
| Isthmus | Splenium | -0.00007 | 7.528E-6 | 264 | -9.42 | <.0001 | <.0001 |
|  |  |  |  |  |  |  |  |

**Supplemental Table 5.** Mixed repeated measures results for between-group differences in MD across CC segments followed by Tukey-Kramer correction for multiple comparisons.

| **CC Segment** | **Estimate** | **Standard Error** | **DF** | **t Value** | **Adj p** |
| --- | --- | --- | --- | --- | --- |
| Rostrum | 7.971E-6 | 0.000019 | 264 | 0.42 | 0.6722 |
| Genu | 0.000010 | 0.000019 | 264 | 0.56 | 0.5775 |
| Rostral Body | -8.12E-6 | 0.000019 | 264 | -0.44 | 0.6613 |
| Anterior Midbody | 0.000027 | 0.000019 | 264 | 1.46 | 0.1452 |
| Posterior Midbody | 0.000046 | 0.000019 | 264 | 2.46 | 0.0145 |
| Isthmus | 7.971E-6 | 0.000019 | 264 | 0.42 | 0.6722 |
| Splenium | 0.000010 | 0.000019 | 264 | 0.56 | 0.5775 |

**Supplemental Table 6.** Mixed repeated measures results for AD differences across CC segments collapsed across groups followed by Tukey Kramer correction for multiple comparisons.

| **CC segment** | **CC segment** | **Estimate** | **Standard Error** | **DF** | **t Value** | **Pr > \|t\|** | **Adj p** |
| --- | --- | --- | --- | --- | --- | --- | --- |
| Rostrum | Genu | 7.502E-6 | 0.000013 | 270 | 0.59 | 0.5527 | 0.9969 |
| Rostrum | Rostral Body | -0.00002 | 0.000013 | 270 | -1.46 | 0.1462 | 0.7698 |
| Rostrum | Anterior Midbody | -0.00008 | 0.000013 | 270 | -6.02 | <.0001 | <.0001 |
| Rostrum | Posterior Midbody | -0.00011 | 0.000013 | 270 | -8.74 | <.0001 | <.0001 |
| Rostrum | Isthmus | -0.00008 | 0.000013 | 270 | -5.97 | <.0001 | <.0001 |
| Rostrum | Splenium | -0.00025 | 0.000013 | 270 | -19.62 | <.0001 | <.0001 |
| Genu | Rostral Body | -0.00003 | 0.000013 | 270 | -2.05 | 0.0411 | 0.3842 |
| Genu | Anterior Midbody | -0.00008 | 0.000013 | 270 | -6.65 | <.0001 | <.0001 |
| Genu | Posterior Midbody | -0.00012 | 0.000013 | 270 | -9.39 | <.0001 | <.0001 |
| Genu | Isthmus | -0.00008 | 0.000013 | 270 | -6.60 | <.0001 | <.0001 |
| Genu | Splenium | -0.00026 | 0.000013 | 270 | -20.34 | <.0001 | <.0001 |
| Rostral Body | Anterior Midbody | -0.00006 | 0.000013 | 270 | -4.50 | <.0001 | 0.0002 |
| Rostral Body | Posterior Midbody | -0.00009 | 0.000013 | 270 | -7.16 | <.0001 | <.0001 |
| Rostral Body | Isthmus | -0.00006 | 0.000013 | 270 | -4.42 | <.0001 | 0.0003 |
| Rostral Body | Splenium | -0.00023 | 0.000013 | 270 | -17.90 | <.0001 | <.0001 |
| Anterior Midbody | Posterior Midbody | -0.00003 | 0.000013 | 270 | -2.63 | 0.0091 | 0.1223 |
| Anterior Midbody | Isthmus | 1.652E-6 | 0.000013 | 270 | 0.13 | 0.8966 | 1.0000 |
| Anterior Midbody | Splenium | -0.00017 | 0.000013 | 270 | -13.44 | <.0001 | <.0001 |
| Posterior Midbody | Isthmus | 0.000035 | 0.000013 | 270 | 2.79 | 0.0056 | 0.0811 |
| Posterior Midbody | Splenium | -0.00014 | 0.000013 | 270 | -10.95 | <.0001 | <.0001 |
| Isthmus | Splenium | -0.00017 | 0.000013 | 270 | -13.74 | <.0001 | <.0001 |

**Supplemental Table 7.** Mixed repeated measures results for RD differences across CC segments collapsed across groups followed by Tukey Kramer correction for multiple comparisons.

| **CC segment** | **CC segment** | **Estimate** | **Standard Error** | **DF** | **t Value** | **Pr > \|t\|** | **Adj p** |
| --- | --- | --- | --- | --- | --- | --- | --- |
| Rostrum | Genu | 0.000018 | 6.878E-6 | 264 | 2.60 | 0.0097 | 0.1289 |
| Rostrum | Rostral Body | 0.000044 | 7.06E-6 | 264 | 6.21 | <.0001 | <.0001 |
| Rostrum | Anterior Midbody | 0.000061 | 6.994E-6 | 264 | 8.67 | <.0001 | <.0001 |
| Rostrum | Posterior Midbody | 0.000038 | 6.878E-6 | 264 | 5.60 | <.0001 | <.0001 |
| Rostrum | Isthmus | 0.000020 | 6.878E-6 | 264 | 2.91 | 0.0039 | 0.0590 |
| Rostrum | Splenium | -4.37E-6 | 6.878E-6 | 264 | -0.64 | 0.5258 | 0.9956 |
| Genu | Rostral Body | 0.000026 | 7.004E-6 | 264 | 3.71 | 0.0003 | 0.0047 |
| Genu | Anterior Midbody | 0.000043 | 6.939E-6 | 264 | 6.15 | <.0001 | <.0001 |
| Genu | Posterior Midbody | 0.000021 | 6.824E-6 | 264 | 3.02 | 0.0028 | 0.0441 |
| Genu | Isthmus | 2.114E-6 | 6.824E-6 | 264 | 0.31 | 0.7570 | 0.9999 |
| Genu | Splenium | -0.00002 | 6.824E-6 | 264 | -3.27 | 0.0012 | 0.0209 |
| Rostral Body | Anterior Midbody | 0.000017 | 7.103E-6 | 264 | 2.35 | 0.0193 | 0.2225 |
| Rostral Body | Posterior Midbody | -5.39E-6 | 7.004E-6 | 264 | -0.77 | 0.4426 | 0.9877 |
| Rostral Body | Isthmus | -0.00002 | 7.004E-6 | 264 | -3.41 | 0.0008 | 0.0133 |
| Rostral Body | Splenium | -0.00005 | 7.004E-6 | 264 | -6.89 | <.0001 | <.0001 |
| Anterior Midbody | Posterior Midbody | -0.00002 | 6.939E-6 | 264 | -3.19 | 0.0016 | 0.0266 |
| Anterior Midbody | Isthmus | -0.00004 | 6.939E-6 | 264 | -5.85 | <.0001 | <.0001 |
| Anterior Midbody | Splenium | -0.00006 | 6.939E-6 | 264 | -9.36 | <.0001 | <.0001 |
| Posterior Midbody | Isthmus | -0.00002 | 6.824E-6 | 264 | -2.71 | 0.0073 | 0.1008 |
| Posterior Midbody | Splenium | -0.00004 | 6.824E-6 | 264 | -6.28 | <.0001 | <.0001 |
| Isthmus | Splenium | -0.00002 | 6.824E-6 | 264 | -3.57 | 0.0004 | 0.0075 |
|  |  |  |  |  |  |  |  |

**Supplemental Table 8.** Mixed repeated measures results for between-group differences in RD across CC segments followed by Tukey-Kramer correction for multiple comparisons.

| **CC Segment** | **Estimate** | **Standard Error** | **DF** | **t Value** | **Adj p** |
| --- | --- | --- | --- | --- | --- |
| Rostrum | 8E-6 | 0.000016 | 264 | 0.50 | 0.6203 |
| Genu | 0.000019 | 0.000016 | 264 | 1.18 | 0.2391 |
| Rostral Body | 6.476E-6 | 0.000016 | 264 | 0.40 | 0.6918 |
| Anterior Midbody | 9.752E-6 | 0.000016 | 264 | 0.60 | 0.5481 |
| Posterior Midbody | 9.919E-7 | 0.000016 | 264 | 0.06 | 0.9507 |
| Isthmus | 0.000035 | 0.000016 | 264 | 2.21 | 0.0281 |
| Splenium | 0.000054 | 0.000016 | 264 | 3.35 | 0.0009 |

**Supplemental Table 9.** Spearman partial correlation results between CC segment diffusion measures and FCVIQ total and factor scores adjusting for age, sex, and preterm birth. Significant correlations at p < 0.05 are denoted in bold font. Imp = impairment

| **Diffusion Metric** | **CC Segment** |  | **FCVIQ total** | **Object face processing imp** | **Visual disinterest** | **Clutter distance viewing imp** | **Moving in space imp** | **Anxiety related behaviour** |
| --- | --- | --- | --- | --- | --- | --- | --- | --- |
| **FA** | **Rostrum** | R  p | 0.08151  0.7818 | -0.05361  0.8556 | 0.19298  0.5086 | -0.03764  0.8984 | 0.10939  0.7097 | -0.06069  0.8367 |
|  | **Genu** | R  p | 0.10509  0.7093 | -0.01523  0.9570 | 0.22178  0.4270 | 0.26905  0.3322 | 0.42197  0.1172 | 0.10736  0.7033 |
|  | **Rostral Body** | R  p | 0.15189  0.6375 | -0.16526  0.6077 | 0.48867  0.1069 | 0.16704  0.6038 | 0.28631  0.3670 | 0.06727  0.8355 |
|  | **Anterior Midbody** | R  p | 0.24773  0.4145 | 0.11185  0.7160 | 0.11933  0.6978 | 0.20277  0.5064 | 0.37439  0.2076 | 0.46428  0.1100 |
|  | **Posterior Midbody** | R  p | 0.14467  0.6070 | -0.20413  0.4656 | 0.08910  0.7522 | 0.01710  0.9518 | 0.29423  0.2871 | 0.12324  0.6617 |
|  | **Isthmus** | R  p | -0.10040  0.7218 | -0.31373  0.2548 | -0.02372  0.9331 | -0.05411  0.8481 | 0.11907  0.6725 | -0.05675  0.8408 |
|  | **Splenium** | R  p | 0.01542  0.9565 | -0.25296  0.3630 | 0.14345  0.6100 | -0.06797  0.8098 | -0.00569  0.9840 | -0.20595  0.4615 |
| **AD** | **Rostrum** | R  p | -0.09772  0.7396 | 0.18695  0.5222 | ***0.58582***  ***0.0277*** | -0.00744  0.9799 | -0.11965  0.6837 | 0.06183  0.8337 |
|  | **Genu** | R  p | -0.39680  0.1431 | -0.22485  0.4204 | -0.24914  0.3706 | -0.05061  0.8579 | -0.39584  0.1441 | -0.23439  0.4004 |
|  | **Rostral Body** | R  p | -0.37944  0.2238 | -0.27239  0.3917 | -0.20080  0.5315 | -0.25901  0.4163 | ***-0.61291***  ***0.0341**** | -0.23282  0.4665 |
|  | **Anterior Midbody** | R  p | -0.23592  0.4378 | -0.37142  0.2115 | -0.34952  0.2417 | 0.03967  0.8976 | -0.50515  0.0783 | -0.27389  0.3652 |
|  | **Posterior Midbody** | R  p | -0.41547  0.1235 | ***-0.57251***  ***0.0257**** | -0.23352  0.4022 | -0.25712  0.3549 | ***-0.54136***  ***0.0371**** | ***-0.52492***  ***0.0445**** |
|  | **Isthmus** | R  p | -0.19442  0.4875 | -0.24879  0.3713 | -0.06381  0.8213 | -0.06211  0.8260 | -0.39206  0.1484 | -0.17259  0.5385 |
|  | **Splenium** | R  p | -0.05385  0.8488 | -0.34401  0.2093 | 0.11674  0.6786 | -0.00697  0.9803 | -0.39622  0.1437 | -0.02938  0.9172 |
| **MD** | **Rostrum** | R  p | -0.10277  0.7266 | 0.20111  0.4906 | 0.32107  0.2630 | 0.04303  0.8839 | -0.22352  0.4424 | 0.06317  0.8301 |
|  | **Genu** | R  p | -0.34992  0.2011 | -0.16995  0.5448 | -0.36460  0.1815 | -0.15085  0.5915 | -0.32546  0.2365 | -0.20926  0.4542 |
|  | **Rostral Body** | R  p | -0.20947  0.5135 | -0.15343  0.6340 | -0.35573  0.2564 | -0.13117  0.6845 | -0.45976  0.1326 | -0.13569  0.6741 |
|  | **Anterior Midbody** | R  p | -0.24695  0.4160 | -0.30792  0.3061 | -0.36691  0.2175 | -0.00727  0.9812 | -0.51813  0.0697 | -0.33128  0.2689 |
|  | **Posterior Midbody** | R  p | -0.34617  0.2063 | -0.29998  0.2774 | -0.36932  0.1755 | -0.19811  0.4791 | ***-0.52855***  ***0.0428**** | -0.44106  0.0998 |
|  | **Isthmus** | R  p | -0.12325  0.6617 | -0.08123  0.7735 | -0.15208  0.5885 | -0.02564  0.9277 | -0.36678  0.1787 | -0.06240  0.8252 |
|  | **Splenium** | R  p | -0.27320  0.3245 | -0.36511  0.1809 | -0.06327  0.8228 | -0.17973  0.5215 | -0.46467  0.0810 | -0.04190  0.8821 |
| **RD** | **Rostrum** | R  p | -0.05318  0.8567 | 0.20559  0.4807 | 0.18573  0.5250 | 0.17840  0.5417 | -0.21477  0.4609 | 0.07210  0.8065 |
|  | **Genu** | R  p | -0.22530  0.4195 | -0.08562  0.7616 | -0.31674  0.2501 | -0.16829  0.5488 | -0.20355  0.4669 | -0.16412  0.5589 |
|  | **Rostral Body** | R  p | -0.19036  0.5534 | -0.08867  0.7841 | -0.48772  0.1077 | -0.10179  0.7529 | -0.49374  0.1028 | -0.17903  0.5777 |
|  | **Anterior Midbody** | R  p | -0.13604  0.6577 | -0.18417  0.5470 | -0.32679  0.2758 | 0.10247  0.7391 | -0.41971  0.1534 | -0.34954  0.2417 |
|  | **Posterior Midbody** | R  p | -0.32854  0.2319 | -0.09053  0.7483 | -0.22634  0.4173 | -0.19963  0.4756 | ***-0.53233***  ***0.0411**** | -0.32000  0.2449 |
|  | **Isthmus** | R  p | -0.06829  0.8089 | 0.00023  0.9993 | -0.02995  0.9156 | -0.11077  0.6943 | -0.32126  0.2430 | -0.01375  0.9612 |
|  | **Splenium** | R  p | -0.31268  0.2565 | -0.23874  0.3915 | -0.16180  0.5646 | -0.25551  0.3580 | -0.39255  0.1478 | 0.05920  0.8340 |
